# Preventing Differentiation Towards Primitive Macrophages in Stem Cells With Down Syndrome

**DOI:** 10.1111/imm.70070

**Published:** 2025-11-27

**Authors:** Koki Harada, Keiichi Ishihara, Sayaka Wakayama, Teruhiko Wakayama, Sae Yamamoto, Haruhiko Sago, Shun Shimohama, Satoshi Akiba, Florent Ginhoux, Kazuyuki Takata

**Affiliations:** ^1^ Joint Research Laboratory, Division of Integrated Pharmaceutical Sciences Kyoto Pharmaceutical University Kyoto Japan; ^2^ Laboratory of Pathological Biochemistry, Division of Pathological Sciences Kyoto Pharmaceutical University Kyoto Japan; ^3^ Advanced Biotechnology Center University of Yamanashi Yamanashi Japan; ^4^ Sanno Birth Center Minato‐ku Tokyo Japan; ^5^ Department of Neurology Sapporo Medical University, School of Medicine Sapporo Japan; ^6^ Jiseikai Hikarigaoka Hospital Nerima Japan; ^7^ Singapore Immunology Network (SIgN), Agency for Science Technology and Research (A*STAR) Singapore Singapore; ^8^ Shanghai Institute of Immunology Shanghai Jiao Tong University School of Medicine Shanghai China; ^9^ Translational Immunology Institute, SingHealth/Duke‐NUS, Academic Medical Center The Academia Singapore Singapore; ^10^ Gustave Roussy Cancer Campus Villejuif France; ^11^ Institut National de la Santé et de la Recherche Médicale (INSERM) U1015 Equipe Labellisée—Ligue Nationale Contre le Cancer Villejuif France

**Keywords:** differentiation, endoderm, mesoderm, microglia, RNA sequencing

## Abstract

Down syndrome (DS) is characterised by delayed brain development and intellectual disabilities. Brain macrophages originate from primitive macrophages that arise from mesodermal cells in the yolk sac and play a role in brain development. Previously, we showed a reduced number of brain macrophages in Ts1Cje foetuses, a murine model of DS. However, the mechanism underlying this reduction in Ts1Cje embryos remains unknown. First, in this study, we identified that the reduced brain macrophages in Ts1Cje foetuses were microglia. This study further reports the low production of primitive macrophages from Ts1Cje mouse embryonic stem cells (Ts1Cje‐mESCs). Gene expression profiling in Ts1Cje‐mESC‐derived cells indicated the persistence of pluripotency, preference for endodermal differentiation, and suppression of mesodermal differentiation. Consistently, Ts1Cje‐mESC‐derived cells showed the disturbed expression of cytokine receptors and apoptosis, and a decreased number of primitive macrophages was confirmed in the Ts1Cje yolk sac. A human induced pluripotent stem cell line established from an individual with DS also showed lower primitive macrophage production relative to diploidized controls. Thus, our data indicate resistance to mesodermal differentiation and impaired production of primitive macrophages in DS, providing valuable insights into abnormalities in brain development associated with DS.

## Graphical abstract

Decreased fetal brain macrophages were previously reported in Ts1Cje mice, a model of Down syndrome (DS). We confirmed the reduction in primitive macrophages, brain macrophage precursors, using Ts1Cje‐derived mouse embryonic stem cells (Ts1Cje‐mESCs) and human induced pluripotent stem cells with trisomy 21 (Tri21‐hiPSCs), with in vivo validation. Ts1Cje‐mESCs preferred endodermal differentiation, and primitive macrophages from Ts1Cje‐mESCs expressed pro‐inflammatory‐related genes. Ts1Cje‐mESCs and Tri21‐hiPSC‐derived cells showed increased apoptosis relative to wild‐type mESCs and hiPSCs with disomy 21 (Di21‐hiPSCs). However, long‐term culture of Tri21‐hiPSC‐derived primitive macrophages revealed that this reduction was transient. These findings improve our understanding of developmental brain abnormalities in DS.

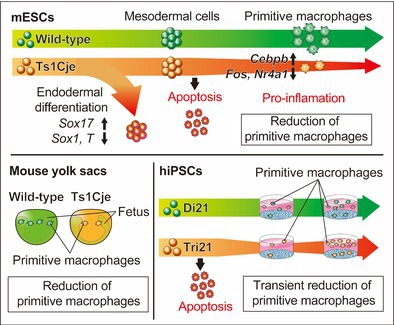

## 1 Introduction

Down syndrome (DS) is a typical aneuploidy caused by whole or partial trisomy of the human chromosome 21 (HSA21), and most individuals with DS show intellectual disability. An analysis of postmortem brains from individuals with DS revealed impaired neurogenesis during prenatal stages [1]. This has led to the widely accepted hypothesis that intellectual disability associated with DS begins in utero because of impaired neurogenesis during early brain development [2].

Primitive macrophages invade the brain primordium before forming the blood–brain barrier and settle as brain macrophages [3, 4]. Brain macrophages, particularly microglia, modulate neurogenesis [5] and neural circuits during brain development [6]. Thus, abnormalities in brain macrophages may be strongly associated with abnormal neurogenesis. We found a decrease in the number of brain macrophages and disturbed neurogenesis in a mouse model of DS [7]. Brain macrophages are composed of microglia in the brain parenchyma and non‐parenchymal brain macrophages, also known as border‐associated macrophages [8]. Brain macrophages originate primarily from mesodermal cells in the yolk sac during the embryonic period [3, 8, 9].

Mouse models are widely used in DS research to understand the pathological mechanisms underlying the various features of DS. The distal end of mouse chromosome 16 (MMU16), which contains approximately 115 genes, is a syntenic region of a large portion of HSA21. The Ts1Cje mouse, one of the widely used mouse models of DS, carries a partial trisomic region of MMU16, which encodes approximately 70 genes, and shows decreased brain volume with impaired cortical neurogenesis in the prenatal brain [10], which recapitulates the features of DS. In the fetal brain of Ts1Cje mice, we previously observed a decrease in the number of brain macrophages [7]. However, the underlying mechanism for such brain macrophage reduction in Ts1Cje embryos remains unknown, and it is also not known whether such a reduction is also observed during the development of human individuals with DS.

One possible reason for the reduced number of brain macrophages is that the trisomic DS genes disrupt the developmental process of primitive macrophages. Recent advancements in stem cell technology have enabled the production of macrophages [11], especially primitive macrophages [12], allowing the analysis of their developmental processes. Therefore, this study analysed abnormalities in the process of differentiation into primitive macrophages using Ts1Cje mouse embryonic stem cells (Ts1Cje‐mESCs) and human induced pluripotent stem cells (hiPSCs) established from an individual with DS. Our results revealed that triplicated genes lead to alterations in gene expression profiles, suggesting persistence of pluripotency, a preference for endodermal differentiation, and suppression of mesodermal differentiation, which may result in reduced numbers of primitive macrophages in DS.

## 2 Materials

### 2.1 Mice

Ts1Cje mice were maintained by mating carrier males with C57BL/6J (The Jackson Laboratory, JAX stock #000664) females. Genotyping of Ts1Cje mice was performed by PCR [13, 14]. Mouse embryos were obtained by mating Ts1Cje male mice with C57BL/6J female mice. Mice were housed with no more than five animals per cage and had ad libitum access to food and water on a 12 h light/dark cycle. All experimental procedures were performed according to the guidelines of the Animal Experiments Committee of Kyoto Pharmaceutical University (approval no. A22‐013).

### 2.2 Establishment of Ts1Cje‐mESCs

To establish mESCs, blastocysts were obtained from naturally mating Ts1Cje males with C57BL/6J females. Blastocysts were obtained by slaughtering pregnant female mice on the second day after mating, collecting two‐cell‐stage embryos from the oviduct, and culturing them to the blastocyst stage. In order to establish mESCs, blastocysts were transferred to 96‐well dishes with feeder cells and cultured for approximately 7–10 days in Glasgow minimal essential medium (GMEM; Thermo Fisher Scientific, Waltham, MA, USA) contained with 20% Knock‐out Serum Replacement (KSR; Invitrogen, Carlsbad, CA, USA), 0.1 mg/mL ACTH (fragments 1–24; Bachem, Bubendorf, Switzerland), non‐essential amino acid solution (NEAA, 1×, FUJIFILM Wako Pure Chemical Corporation, Osaka, Japan), 2‐mercaptoethanol solution (1×, FUJIFILM Wako Pure Chemical Corporation), pyruvic acid (1×, FUJIFILM Wako Pure Chemical Corporation), and 1.0 × 10^3^ units/mL of mouse LIF (FUJIFILM Wako Pure Chemical Corporation) [15]. mESCs were maintained on feeder‐free 0.1% gelatin (Sigma‐Aldrich, St. Louis, MO, USA)‐coated plates filled with KSR medium [KnockOut DMEM (Thermo Fisher Scientific) containing 20% KSR, 2.0 mM glutamine (FUJIFILM Wako Pure Chemical Corporation), NEAA (1×), 2‐mercaptoethanol solution (1×), penicillin–streptomycin solution (1×, FUJIFILM Wako Pure Chemical Corporation) and 1.0 × 10^3^ units/mL of mouse LIF] in an incubator set at 37°C, under 5% CO_2_ and 95% air. mESCs were confirmed to be free from mycoplasma contamination.

### 2.3 hiPSCs Culture

The hiPSCs from an individual with DS (HPS4270) and isogenic hiPSCs with disomy 21 (HPS4272) were recently provided by the RIKEN BRC Cell Bank through the National BioResource Project of MEXT, Japan [16]. hiPSCs underwent short tandem repeat profiling at the RIKEN BRC and were confirmed to be free from mycoplasma contamination both before and after being provided. hiPSCs were maintained on iMatrix‐511 silk (Matrixome, Osaka, Japan)—coated plates filled with essential 8 medium (Thermo Fisher Scientific) in an incubator set at 37°C and 5% CO_2_. The medium was changed daily, and the cells were replated once per week. The experiments with hiPSCs were approved by the Ethical Review Committee for Medical and Health Research Involving Human Subjects at Kyoto Pharmaceutical University (Approval No. 20‐18‐26).

### 2.4 G‐Band Karyotyping

G‐band karyotyping of cell lines was performed by Nihon Gene Research Laboratories (Sendai, Japan). In brief, the mESCs and hiPSCs were treated with colcemid to enrich metaphase cells, detached using trypsin, exposed to buffered hypotonic solution, and fixed thrice with 3:1 methanol: acetic acid at room temperature. Fixed cells were detached with trypsin and subsequently stained with Giemsa solution. Karyotypes were analysed in 20 cells for each cell line. Karyotyping of Ts1Cje‐mESCs and hiPSCs with trisomy 21 confirmed a translocation of mouse chromosome 16 to mouse chromosome 12 and triplication of human chromosome 21, respectively (Figure S1A,B).

### 2.5 Generation of Primitive Macrophages From mESCs

mESC‐derived primitive macrophages were generated according to a previously reported protocol [12]. Serum‐free differentiation (SF‐DIFF) medium was prepared by filtration with Vacuum Filtration rapid‐Filtermax (TPP Techno Plastic Products AG, Trasadingen, Switzerland) after mixing IMDM (Thermo Fisher Scientific) and Ham's F‐12 medium (Thermo Fisher Scientific) at a ratio of 3:1, and N2 supplement (1×, Thermo Fisher Scientific), AscleStem Neuronal Supplement (1×, Nacalai Tesque, Kyoto, Japan) or B‐27 supplement (1×, Thermo Fisher Scientific), 0.1% bovine serum albumin (BSA, Fujifilm Wako Chemicals), penicillin–streptomycin (1×), and 3.0 mM glutamine were added. On days 0, 2, and 4 of differentiation, cells were cultured in SF‐DIFF medium and further supplemented with the following cytokines and chemicals: day 0: 0.5 mM L‐ascorbic acid (Sigma‐Aldrich), 0.45 mM monothioglycerol (Fujifilm Wako Chemicals), 5 ng/mL recombinant human bone morphogenic protein‐4 (rhBMP‐4, R&D Systems, Minneapolis, MN, USA), 5 ng/mL recombinant human fibroblast growth factor 2 (rhFGF2, R&D Systems); day 2: 0.5 mM L‐ascorbic acid, 0.45 mM monothioglycerol, 2 ng/mL rhBMP‐4, 5 ng/mL rhFGF2, 5 ng/mL recombinant human vascular endothelial growth factor (rhVEGF, R&D Systems), and 2 ng/mL recombinant human/mouse/rat activin A (rhactivin A, R&D Systems); day 4: 0.5 mM L‐ascorbic acid, 0.45 mM monothioglycerol, 25 μg/mL gentamicin (FUJIFILM Wako Pure Chemical Corporation), 5 ng/mL rhVEGF and 100 ng/mL recombinant human colony stimulating factor 1 (rhCSF‐1, R&D Systems). On day 6, the culture medium was switched to StemPro‐34 medium (Thermo Fisher Scientific) containing 0.5 mM L‐ascorbic acid, 0.45 mM monothioglycerol, penicillin–streptomycin (1×), 150 μg/mL transferrin from human serum (Roche, Basel, Switzerland), 2.0 mM glutamine, 50 ng/mL rhCSF‐1, 100 ng/mL recombinant human stem cell factor (rhSCF, Nacalai Tesque), 10 ng/mL recombinant human interleukin‐3 (rhIL‐3, R&D Systems). mESCs or embryonic bodies were dissociated in TrypLE (1×, Thermo Fisher Scientific) for 4 min and collected by centrifugation at 200 × g for 5 min using a table‐top centrifuge (Model2420, KUBOTA, Tokyo, Japan). For terminal differentiation towards primitive macrophages, collected cells were seeded with SF‐DIFF (days 0, 2, and 4) or StemPro medium (day 6) into non‐coated dishes (days 0, 2, and 6) and poly (2‐hydroxyethyl methacrylate) (HEMA, Sigma‐Aldrich)‐coated plates (day 4) at 2.0–5.0 × 10^5^ cells/mL and incubated under 5% CO_2_ at 37°C.

### 2.6 Generation of Primitive Macrophages From hiPSCs

hiPSC‐derived primitive macrophages were differentiated according to previously published protocols [12]. Two days before the start of differentiation, hiPSCs were dissociated in TrypLE (0.5×) for 8 min, collected by centrifugation at 200 × g for 5 min, and seeded at a density of 3.3 × 10^3^ cells/cm^2^ in essential 8 medium supplemented with 10 μM Y‐27632 (10 μM, Selleck Chemicals, Houston, TX, USA). Cells were cultured in StemPro‐34 medium mixed with 150 μg/mL human serum‐derived transferrin, 2.0 mM glutamine, 500 μM L‐ascorbic acid, 0.45 mM monothioglycerol and penicillin–streptomycin (1×). In addition, the following cytokines and chemicals were added to the medium at each schedule and the medium was changed every 2 days: day 0, 5 ng/mL rhBMP‐4; 50 ng/mL rhVEGF and 2 μM CHIR99021 (Selleck Chemicals); day 2, 5 ng/mL rhBMP‐4, 50 ng/mL rhVEGF, and 20 ng/mL rhFGF2; day 4, 15 ng/mL rhVEGF, and 5 ng/mL rhFGF2; days 6–10, 10 ng/mL rhVEGF, 10 ng/mL rhFGF2, 50 ng/mL rhSCF, 30 ng/mL recombinant human dickkopf‐1 (hrDKK‐1, R&D Systems), 10 ng/mL recombinant human interleukin‐6 (rhIL‐6; R&D Systems), and 10 ng/mL rhIL‐3; day 12–14, 10 ng/mL rhFGF 2, 50 ng/mL rhSCF, 10 ng/mL rhIL‐6, 10 ng/mL rhIL‐3. After day 16, hiPSC‐derived cells were cultured in SF‐DIFF medium containing 30 ng/mL rhCSF‐1 and the medium was changed every 2–3 days. From days 0 to 7, cells were grown under a hypoxic atmosphere (37°C, 5% CO_2_, 5% O_2_) and cultured under normoxia from day 8 onwards (37°C, 5% CO_2_); from day 6 onwards, suspended cells were collected and brought back to the original culture dish with fresh medium.

### 2.7 Quantification of the Cell Number

The mESCs and hiPSCs were detached and dissociated into single cells using TrypLE (1×), and the number of cells was counted using a haemocytometer (ERMA Inc., Saitama, Japan). Equal numbers of cells were seeded in culture dishes. On differentiation days 0, 2, 4, 6, 12, and 30, live cells were counted using a haemocytometer after Trypan Blue (Nacalai Tesque) staining.

### 2.8 Embryoid Bodies Formation Assay

Cytokine‐free medium for EB assay was prepared with GMEM, 8% KSR, 2.0 mM glutamine, NEAA (1×), 2‐mercaptoethanol solution (1×), pyruvic acid (1×) and penicillin–streptomycin solution (1×). mESCs and hiPSCs were cultured at 5.0 × 10^5^ cells in poly‐hema‐coated 6‐well plates with 8% KSR in an incubator set at 37°C, 5% CO_2_, and 95% air, and hiPSCs were treated with 10 μM Y‐27632. These embryoid bodies were centrifuged at 200× *g* for 5 min and resuspended in 8% KSR every 3 days. Total RNA was extracted on day 14.

### 2.9 Flow Cytometry

Fluorescence‐activated cell sorting (FACS) buffer was prepared by filtering phosphate‐buffered saline (PBS) [2.7 mM KCl (Nacalai Tesque), 1.5 mM KH_2_PO_4_ (Nacalai Tesque), 137 mM NaCl (Nacalai Tesque), 18 mM Na_2_HPO_4_·7H_2_O (Nacalai Tesque)] containing 0.5% BSA and 125 mM di‐sodium dihydrogen ethylenediaminetetraacetate (Nacalai Tesque), and blocking buffer was prepared by adding 1% mouse serum (Sigma‐Aldrich) and 1% rat serum (Sigma‐Aldrich) to the FACS buffer. mESC‐derived macrophage progenitors were detached using TrypLE (1×) for 3 min and then incubated with primary antibodies: anti‐BMPR1B (1:300, R&D Systems, MAB5051), anti‐VEGFR1 (1:8, R&D Systems, AF471), anti‐VEGFR2 (1:8, R&D Systems, AF644), anti‐VEGFR3 (1:80, R&D Systems, AF743), Mouse IgG2B Isotype Control (1:200, R&D Systems, MAB004) and Normal Goat IgG Control (1:40, R&D Systems, AB‐108‐C) in blocking buffer for 20 min at 4°C. The cells were washed by diluting the suspension with 1 mL FACS buffer and centrifugation at 200× *g* for 5 min. The secondary antibodies, Alexa Fluor 647 conjugated anti‐mouse IgG (1:200, Abcam, ab150107) and Alexa Fluor 647 conjugated anti‐goat IgG (1:200, Jackson ImmunoResearch, 805‐607‐008, West Grove, PA, USA), were diluted in blocking buffer and incubated with the cells for 20 min at 4°C. Mouse yolk sac (E8.5) and brain (E14.5) removed surgically from the embryos were incubated in RPMI medium (FUJIFILM Wako Pure Chemical Corporation) containing collagenase (Sigma‐Aldrich), DNase I (Sigma‐Aldrich), and 10% fetal bovine serum (Nichirei Biosciences Inc., Tokyo, Japan) at 37°C for 30 min. The yolk sacs and brains were dissociated into single cells by pipetting. mESC‐ or hiPSC‐derived primitive macrophages were collected by gentle pipetting and centrifugation at 200 × *g* for 5 min. Mouse embryos and mESC‐derived macrophages were stained with APC‐Cy7 rat anti‐mouse CD45 (1:100, BD Bioscience, 557 659, Franklin Lakes, NJ, USA), PE‐Cy7 rat anti‐CD11b (1:200, BD Bioscience, 552 850), APC anti‐mouse F4/80 (1:200, Biolegend, 123 116, San Diego, CA, USA), FITC anti‐Ly‐6G (1:100, Biolegend, 127 605), Pacific Blue anti‐Ly‐6C (1:100, Biolegend, 128 014), and PE anti‐CD206 (1:100, Biolegend, 141 706) in blocking buffer for 20 min at 4°C. hiPSC‐derived macrophages were stained with PE‐Cy7 anti‐human CD45 antibodies (1:200, BioLegend, 304 015) and Alexa Fluor 647 anti‐human CD11b antibodies (1:200, BioLegend, 301 319) in blocking buffer for 20 min at 4°C. To detect apoptotic cells, the cells were stained with PE Annexin V (1:20, BioLegend, 640 907) in Hank's balanced salt solution with calcium (Nacalai Tesque) for 15 min at room temperature in the dark after immunostaining. The cells were stained with 7‐AAD (1:100, Thermo Fisher Scientific, 00‐6993‐05) in FACS buffer, and analysed and sorted using a Fortessa X‐20 (BD Biosciences) or FACS JAZZ (BD Biosciences). The purity of the sorted primitive macrophages (CD45^+^/F4/80^+^) was > 90%. The Kaluza (Beckman Coulter, Brea, CA, USA), was used for further analyses and panel preparation.

### 2.10 Whole‐Mount Immunostaining and Immunofluorescence

E8.5 mouse embryos were fixed in 4% paraformaldehyde (PFA; TAAB Laboratories, Reading, UK) in PBS at 4°C overnight. Fixed mouse embryos were stained with primary antibodies against anti‐GATA1 (1:200, Santa Cruz, sc‐265, Dallas, TX, USA) and anti‐PHH3 (1:400, Thermo Fisher Scientific, PA5‐17869) in 0.2% Triton X (Nacalai Tesque)/PBS (PBST) containing 2% BSA and 5% goat serum (Vector laboratories, S‐1000, Newark, CA, USA) at 4°C overnight. The embryos were washed in 0.2% PBST and then stained with secondary antibodies Alexa Fluor 488 anti‐rabbit IgG (1:250, Invitrogen, A31627) and Alexa Fluor 594 anti‐rat IgG (1:250, Invitrogen, A11007) in 0.2% PBST containing 2% BSA and 5% goat serum at room temperature for 1 h. After staining with 0.2% PBST containing DAPI (1:700; Dojin, Kumamoto, Japan) for 15 min, the yolk sac was opened and mounted on a glass slide.

hiPSC‐derived macrophages were collected from the culture supernatant by centrifugation at 200× *g* for 5 min, resuspended in differentiation medium, and seeded onto glass‐bottom dishes. The cells were then washed with PBS and fixed with 4% PFA/PBS for 30 min at room temperature. The primary antibodies, anti‐Ki67 (1:100, Leica Biosystems, NCL‐L‐Ki67‐MM1, Nussloch, Germany) and anti‐Iba1 (1:1000, FUJIFILM Wako Pure Chemical Corporation, 019–19 741) were diluted in 0.1% PBST containing 5% donkey serum (Jackson ImmunoResearch) and incubated with the fixed cells at 4°C overnight. After washing with 0.1% PBST, the secondary antibodies, Alexa Fluor 488‐conjugated anti‐mouse IgG antibodies (1:500, Thermo Fisher Scientific, A21202) and Alexa Fluor 647‐conjugated anti‐rabbit IgG antibodies (1:500, Thermo Fisher Scientific, A32795), were diluted in 0.1% PBST containing 5% donkey serum and incubated with the fixed cells for 2 h at room temperature in the dark. Hoechst 33342 (1:2000; Dojin) was added to the secondary antibody solution for nuclear staining. Fluorescence was detected using a confocal laser‐scanning microscope (LSM800, Zeiss, Oberkochen, Germany), and analysed using Zen software (Zeiss) and Image J [17].

### 2.11 RNA‐Seq

Total RNA (OD260/280 > 1.8) was extracted from mESC‐derived cells on differentiation days 4 and 12 using a Nucleospin RNA kit and Nucleospin RNA XS kit (Macherey‐Nagel, Düren, Germany), respectively. The quality of total RNA on differentiation day 4 was evaluated using an Agilent 2100 Bioanalyzer (Agilent, Santa Clara, CA, USA) (RNA integrity number value > 9.0), and a cDNA sequencing library was prepared using the NEBNext Poly(A) mRNA Magnetic Isolation Module (New England Biolabs, Ipswich, MA, USA) and NEBNext Ultra II Directional RNA Library Prep Kit (New England Biolabs). Because of the small amount of total RNA, a library of total RNA on differentiation day 12 was prepared using the SMART‐seq HT Plus kit (Takara Bio, Shiga, Japan). RNA‐Seq was performed in paired‐end 150 bp mode using a NovaSeq 6000 sequencer (Illumina, San Diego, CA, USA) by Rhelixa Co. Ltd. (Tokyo, Japan).

### 2.12 Analyses of RNA‐Seq Data

Low‐quality bases and adapter sequences were trimmed, and the data were aligned to the reference genome using the Trimmomatic [18] and HISAT2 [19] software programs, respectively. Read counts were calculated using featureCounts and normalized to transcripts per million (TPM). PCA was performed with RNA‐Seq data, filtered to exclude TPM < 5, using Python 3 (https://www.python.org/) with the Scikit‐learn library [20]. Genes with *p* values of < 0.05 and fold change > 3/2 or < 2/3 were defined as DEGs. GO analyses were performed using DAVID (https://david.ncifcrf.gov/), and clustering, heatmap, and GO analysis results were generated using Python 3 [21, 22]. Volcano plots and expression analyses of trisomic genes were performed in Excel (Microsoft, Washington, DC, USA). We and other groups found the upregulated expression of the *Dnahc11* gene, similar to previous transcriptomic analyses in the present study [7, 23, 24]. It has already been shown that the increased expression in Ts1Cje mice is caused by the truncation of the *Dnahc11* gene by the translocation of the trisomic region to the distal end of Mmu12 resulting in the overexpression of truncated Dnahc11 [24]; therefore, RNA‐Seq data were analysed excluding *Dnahc11*.

### 2.13 Quantitative Reverse Transcription‐Polymerase Chain Reaction (RT‐qPCR)

Total RNA was extracted from the samples using a NucleoSpin RNA kit. Reverse transcription was performed using the High‐Capacity cDNA Reverse Transcription Kit (Thermo Fisher Scientific). Real‐time PCR was conducted using the PowerUp SYBR Green Master Mix (Thermo Fisher Scientific) on either the LightCycler 96 Instrument (Roche) or CronoSTAR 96 Real‐Time PCR System (WAKENBTECH, Kyoto, Japan). The data were analysed using the *∆*CT method, normalising the raw data to the GAPDH gene. The primer sequences used are listed in Table S1.

### 2.14 Statistical Analysis

Data presented in this study are expressed as the mean ± standard error of the mean (SEM). Outliers were identified and excluded from statistical analysis using Grubbs's test (*α* = 0.05). In the statistical analysis, an analysis of variance (ANOVA) or two‐way ANOVA was performed. The two‐tailed unpaired *t*‐test or two‐tailed one‐sample *t*‐test was used for comparisons between the two groups. In cases with more than three groups, Bonferroni's multiple comparison test was used. Statistical analyses were performed using GraphPad Prism (ver. 6.0 for Windows, GraphPad Software, La Jolla, CA, USA).

## 3 Results

### 3.1 Decreased Generation of Primitive Macrophages From Ts1Cje‐mESCs

**FIGURE 1:**
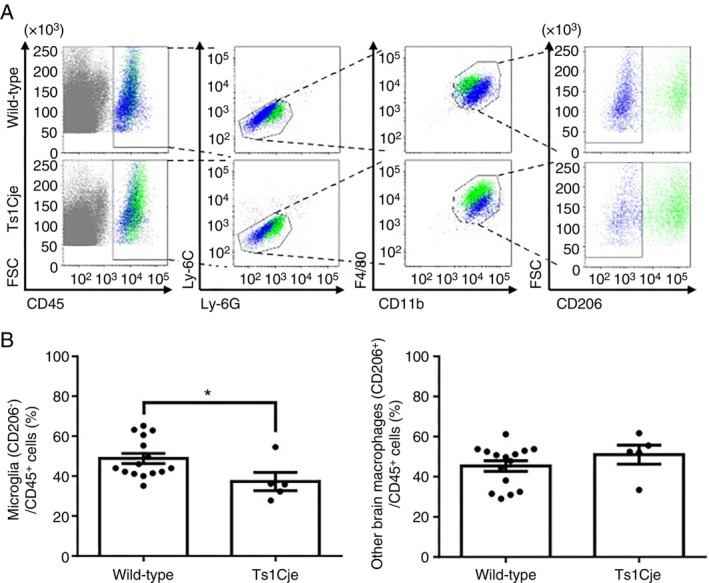
Microglia decreased in the brain of Ts1Cje mice at E14.5. (A) Fetal brains at E14.5 were analysed by flow cytometry using fluorochrome‐labelled antibodies against CD45, Ly‐6C, Ly‐6G, CD11b, F4/80 and CD206. (B) The graphs show the percentages of microglia (left, CD45^+^/Ly‐6C^−^/Ly‐6G^−^/CD11b^+^/F4/80^+^/CD206^−^) and the other brain macrophages (right, CD45^+^/Ly‐6C^−^/Ly‐6G^−^/CD11b^+^/F4/80^+^/CD206^+^) among CD45^+^ cells. Data are presented as the mean ± SEM, *n* = 15 for wild‐type mice and *n* = 5 for Ts1Cje mice. Fetal brains were collected from littermates. Statistical analyses were performed using a two‐tailed unpaired *t*‐test. *p < 0.05 (*p* = 0.0374, left), not significant (*p* = 0.3011, right).

**FIGURE 2:**
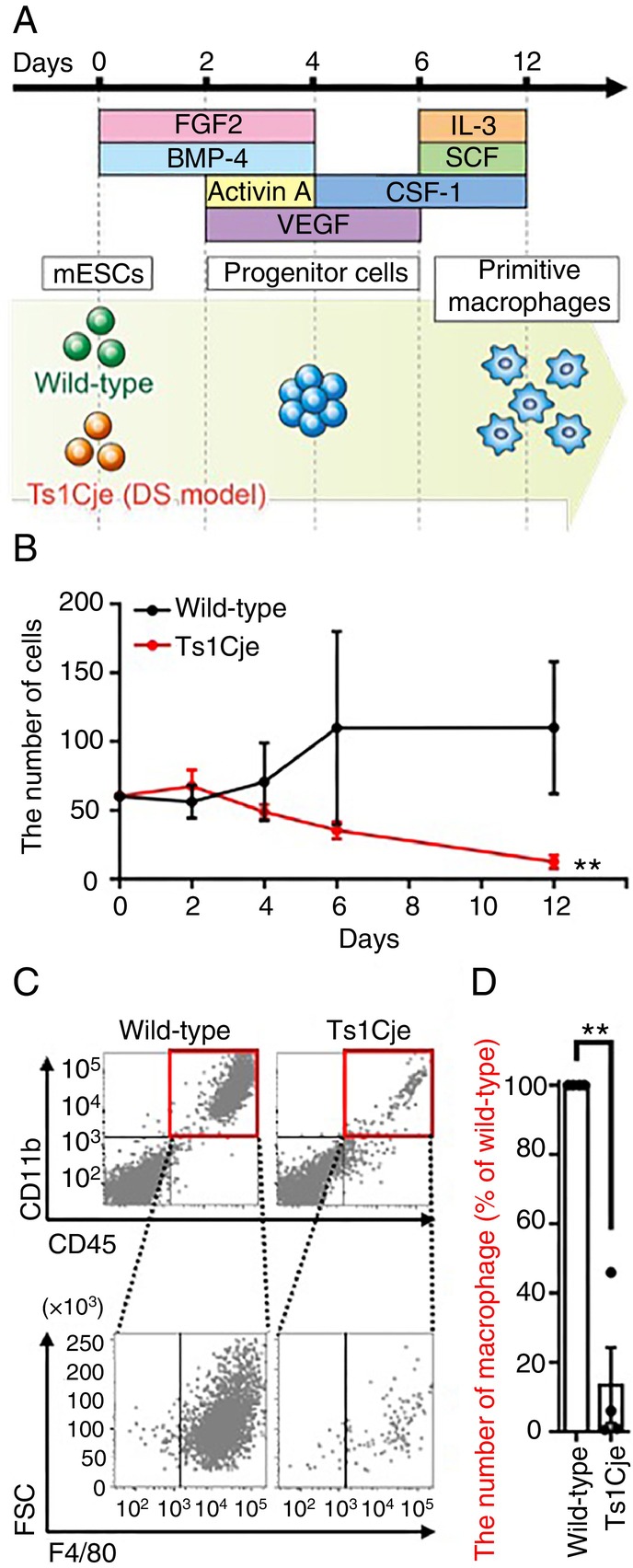
Trisomic genes disturbed differentiation into primitive macrophages from mESCs. (A) Schematic diagram of the differentiation schedule of mESC‐derived primitive macrophages. (B) Decreased cell number of Ts1Cje‐mESC‐derived cells in the process of differentiation into primitive macrophages. The number of wild‐type‐mESC‐ and Ts1Cje‐mESC‐derived cells was counted at each stage of primitive macrophage differentiation (*n* = 4 for each group). Data are presented as the mean ± SEM from four independent biological replicates. Statistical analysis was performed using two‐way ANOVA relative to wild‐type‐mESC‐derived cells. Not significant (*p* = 0.0840 interaction, *p* = 0.09738 days); **p < 0.01 (*p* = 0.026 wild‐type vs. Ts1Cje). (C) A marked decrease in the number of primitive macrophages derived from Ts1Cje‐mESCs. Primitive macrophages (CD45^+^/CD11b^+^/F4/80^+^) derived from wild‐type‐mESCs and Ts1Cje‐mESCs were analysed by flow cytometry and they are shown in the panels. (D) Graphs show the total number of primitive macrophages calculated as a percentage of wild‐type (*n* = 4 for each group). Data are presented as the mean ± SEM from four independent biological replicates. Statistical analysis was performed using a two‐tailed one sample *t*‐test. **p < 0.01 (*p* = 0.0042). BMP‐4, bone morphogenic protein‐4; CSF‐1, colony stimulating factor 1; DS, Down syndrome; FGF2, fibroblast growth factor 2; IL‐3, interleukin‐3; mESCs, mouse embryonic stem cells; SCF, stem cell factor; VEGF, vascular endothelial growth factor.

We previously reported fewer brain macrophages, including microglia, in the fetal brains of Ts1Cje mice than wild‐type mice [7]. To identify which subpopulations of brain macrophages were reduced in Ts1Cje mice, a FACS analysis was performed using an anti‐CD206 antibody (Figure 1A). Brain macrophages can reportedly be distinguished into microglia (CD206^−^) and meningeal macrophages (CD206^+^) based on the CD206 expression [9, 25]. Furthermore, perivascular macrophages have not yet emerged in the fetal brain at E14.5; CD206^−^ brain macrophages are defined as microglia, including those located around blood vessels [25]. As a result, the percentage of microglia (CD45^+^/Ly‐6C^−^/Ly‐6G^−^/CD11b^+^/F4/80^+^/CD206^−^), but not other brain macrophages (CD45^+^/Ly‐6C^−^/Ly‐6G^−^/CD11b^+^/F4/80^+^/CD206^+^), was significantly reduced in fetal brains of Ts1Cje mice at E14.5 compared to wild‐type mice (Figure 1B). Thus, the population of brain macrophages that was decreased in Ts1Cje embryonic brains was identified as microglia. A possible reason for this is that the differentiation process from stem cells to primitive macrophages is impaired; therefore, we evaluated the effects of the triplicated genes in Ts1Cje mice on the differentiation of mESCs into primitive macrophages. For this purpose, a differentiation protocol that recapitulates stepwise fetal haematopoietic processes in the yolk sac to produce primitive macrophages [12] was applied to Ts1Cje‐mESCs (Figure 2A). The differentiation protocol induces cells to adopt a mesodermal fate and normally generates CD45^+^/CD11b^+^/F4/80^+^ macrophages (corresponding to primitive macrophages in vivo) on differentiation day 12 from mouse pluripotent stem cells.

We counted the number of live cells on each differentiation day using a haemocytometer with Trypan Blue exclusion dye. As a result, no difference was shown between the number of wild‐type and Ts1Cje‐mESC‐derived cells on differentiation day 2; however, the number of cells on days 4–6 (corresponding to hemangioblasts in vivo) started to decrease in Ts1Cje‐mESCs in comparison to wild‐type mESCs (Figure 2B). The total number of cells derived from Ts1Cje‐mESCs on day 12 was significantly lower than that derived from wild‐type mESCs (Figure 2B). Next, a FACS analysis using antibodies for CD11b, CD45, and F4/80 was performed to assess the efficacy of the differentiation towards primitive macrophages at differentiation day 12. The percentage of macrophage numbers (CD45^+^/CD11b^+^/F4/80^+^) among the total cell numbers was decreased but not significant in Ts1Cje‐mESCs (38.3% ± 18.1%) in comparison with wild‐type mESCs (57.5% ± 6.4%) (Figure 2C), suggesting that Ts1Cje‐mESCs are able to differentiate towards primitive macrophages if there are the precursor cells. However, the number of primitive macrophages derived from Ts1Cje‐mESCs, adjusted by their percentage of differentiated cells (CD45^+^/CD11b^+^/F4/80^+^) within the live cell on differentiation day 12, was significantly reduced compared to wild‐type (Figure 2D). To confirm the reproducibility of the effect of trisomic genes on primitive macrophage numbers, we performed experiments using multiple mESC lines derived from wild‐type and Ts1Cje mice. As shown in Figure S2, a marked reduction in the number of differentiated primitive macrophages was consistently observed in Ts1Cje‐mESCs compared to wild‐type mESCs. Thus, triplicated genes in Ts1Cje mice decreased the number of mesodermal progenitor cells and resulted in a decreased number of primitive macrophages derived from Ts1Cje‐mESCs in comparison to wild‐type mESCs.

### 3.2 Differentially Expressed Genes in Wild‐Type‐mESC‐ and Ts1Cje‐mESC‐Derived Cells on Differentiation Day 4

**FIGURE 3:**
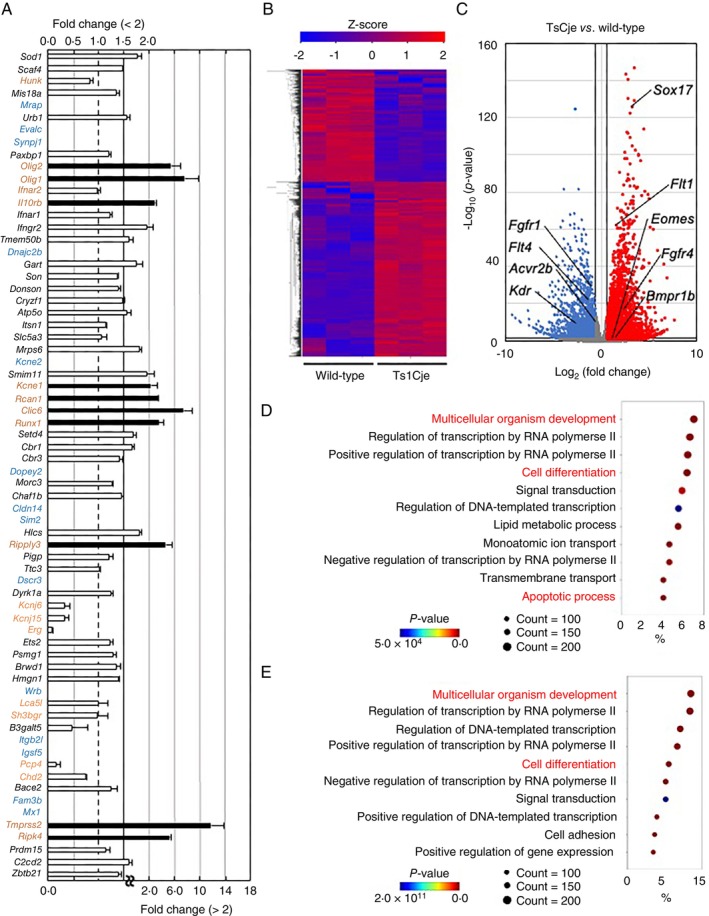
Trisomic genes disturbed differentiation into three germ layers on day 4. (A) Expression change of genes in the trisomic region of Ts1Cje mice. Genes with fold change < 2 are represented by white bars (corresponding to the upper axis), and genes with fold change > 2 are represented by black bars (corresponding to the lower axis). The colours of the genes, black, red, brown and blue, indicate approximately 1.5‐fold change, > 2‐fold upregulation, downregulation, and no expression, respectively (*n* = 3 for each group). Data presented an overview of expression trends as the mean ± SEM from three independent biological replicates. (B) Heatmap of genes with *p* values < 0.05 and fold change > 3/2 or < 2/3 in the RNA‐Seq dataset of wild‐type‐mESC‐ and Ts1Cje‐mESC‐derived cells on differentiation day 4. The colours of the plots, red and blue, indicate significant upregulation and downregulation, respectively (*n* = 3 for each group). Data presented from three independent biological replicates. Statistical analysis was performed using Wald test and adjusted using Benjamini–Hochberg correction. (C) A Volcano plot drawn with the RNA‐Seq dataset. (D, E) The GO analysis of DEGs. The GO enrichment analysis of DEGs upregulated (D) and downregulated (E) in Ts1Cje‐mESC‐derived cells was retrieved using the DAVID program. Top 10 enriched GO terms in biological process are presented (*n* = 3 for each group). Data presented from three independent biological replicates. Statistical analysis was performed using Fisher's exact test.

To understand the effects of triplicated genes in Ts1Cje‐mESCs on primitive macrophage differentiation, we conducted a comprehensive transcript analysis using bulk RNA sequencing (RNA‐Seq). We first analysed cells on differentiation day 4, when no marked difference in the number between the genotypes was detected (Figure 2B). A principal component analysis (PCA) revealed two well‐separated clusters of cells derived from Ts1Cje‐mESCs and wild‐type mESCs (Figure S3). Many triplicated genes in Ts1Cje‐mESC‐derived cells were upregulated in a gene dose‐dependent manner (black text in Figure 3A). In contrast, the expression of the 10 trisomic genes *Olig2*, *Olig1*, *Il10rb*, *Kcne1*, *Rcan1*, *Clic6*, *Runx1*, *Ripply3*, *Tmprss2*, and *Ripk4* was upregulated more than 2.0‐fold in Ts1Cje‐mESC‐derived cells in comparison to that in wild‐type mESC‐derived cells (red text in Figure 3A). Furthermore, the mRNA expression levels of the nine trisomic genes *Hunk*, *Ifnar2*, *Kcnj6*, *Kcnj15*, *Erg*, *Lca5l*, *Sh3bgr*, *Pcp4* and *Chd2*, were decreased in Ts1Cje‐mESC‐derived cells (brown text in Figure 3A), and the transcripts of 14 trisomic genes were undetectable in Ts1Cje‐ or wild‐type‐mESC‐derived cells (blue text in Figure 3A).

A total of 5396 differentially expressed genes (DEGs), defined as genes with *p* values of < 0.05, and fold change of > 3/2 or < 2/3, were detected. The heatmap (Figure 3B) and volcano plot (Figure 3C) showed 3283 upregulated genes and 2113 downregulated genes, respectively. Notably, both the upregulated and downregulated genes include receptors involved in the determination of three germ layers, such as *Flt1*, *Fgfr4*, *Bmpr1b*, and *Fgfr1*, *Flt4*, *Acvr2b* and *Kdr*, respectively [26, 27, 28, 29, 30, 31, 32]. In particular, the expression of endoderm markers, including *Eomes* [33] and *Sox17* [34] was increased in Ts1Cje‐mESC‐derived cells despite the condition for differentiation towards the mesoderm (Figure 3C).

A gene ontology (GO) analysis of both upregulated (Figure 3D) and downregulated genes (Figure 3E) in Ts1Cje‐mESC‐derived cells in comparison to wild‐type mESC‐derived cells indicated gene enrichment in categories including multicellular organism development and cell differentiation. The upregulated genes in Ts1Cje‐mESC‐derived cells further indicated gene enrichment in the apoptotic process category (Figure 3D). It is worth noting that the altered gene expression is found throughout the entirety of the genome and is not only linked to the partial trisomic region of MMU16, which encodes approximately 70 genes.

### 3.3 Expression of Lineage‐Determining Genes in mESC‐Derived Cells on Day 4

**FIGURE 4:**
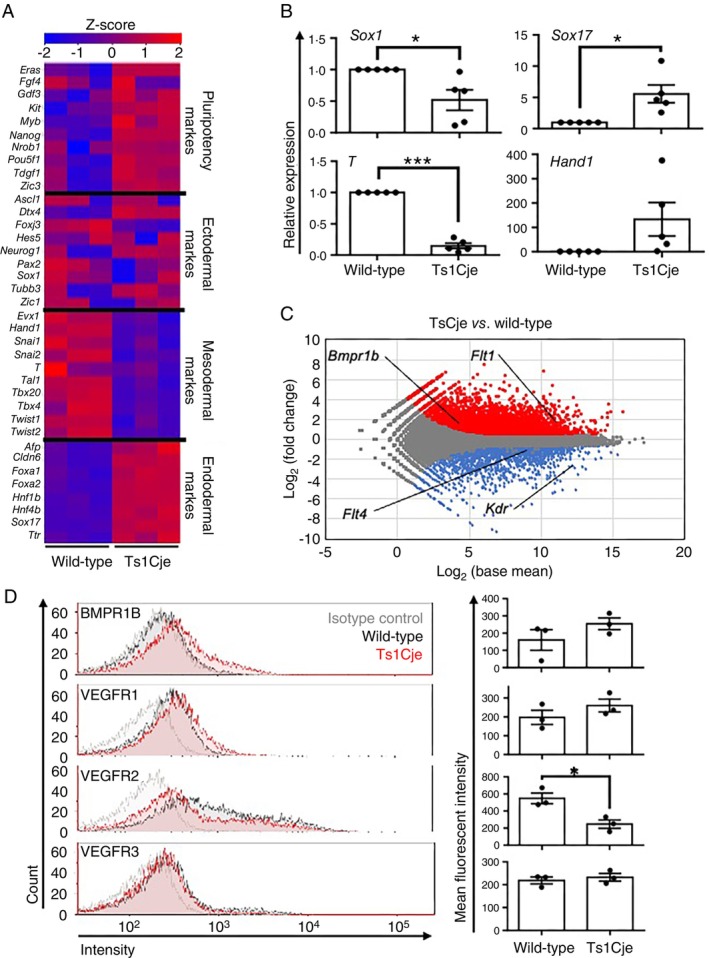
Ts1Cje‐mESC‐derived cells tend to differentiate into endoderm. (A) A heatmap of the expression of genes related to pluripotency and three germ layer markers with RNA‐Seq dataset of wild‐type‐mESC‐ and Ts1Cje‐mESC‐derived cells on differentiation day 4 (*n* = 3 for each group). Data presented an overview of expression trends from three independent biological replicates. (B) Spontaneous differentiation abnormalities in mesodermal and endodermal lineages of Ts1Cje‐mESCs in cytokine‐free medium. Comparison of the gene expression levels between wild‐type‐ and Ts1Cje‐mESC‐derived cells differentiated in an embryonic body for 14 days in cytokine‐free medium (*n* = 5 for each group). Data are presented as the mean ± SEM from five independent biological replicates. Statistical analysis was performed using two‐tailed one sample *t*‐test. Not significant (*p* = 0.1271 *Hand1*); *p < 0.05 (*p* = 0.0416 *Sox1*, *p* = 0.032 *Sox17*); ***p < 0.001 (*p* < 0.0001 *T*). (C) The MA plot was drawn with the RNA‐Seq dataset. In the plots, red and blue, indicate significant upregulation (*p* value < 0.05, fold change > 3/2) and downregulation (*p* value < 0.05, fold change < 3/2), respectively (*n* = 3 for each group). Data presented from three independent biological replicates. Statistical analysis was performed using Wald test and adjusted using Benjamini–Hochberg correction. Annotated genes are cytokine receptors suggesting the relationship to differentiation into mesoderm and endoderm. (D) The disturbed expression of cytokine receptors in Ts1Cje‐mESC‐derived cells. Comparison of the cytokine receptor expression by flow cytometry. Histograms are representative examples of BMPR1B, VEGFR1, VEGFR2 and VEGFR3, respectively (*n* = 3 for each group). Data are presented as the mean ± SEM from three independent biological replicates. Statistical analysis was performed using two‐tailed unpaired *t*‐test. Not significant (*p* = 0.2463 BMPR1B, *p* = 0.2821 VEGFR1, *p* = 0.5714 VEGFR3); *p < 0.05 (*p* = 0.0194 VEGFR2).

Given the increased expression of endoderm markers, such as *E omes* and *S ox 17* (Figure 3C), and the enrichment of genes in categories related to multicellular organism development and cell differentiation (Figure 3D,E), we further focused on the genes associated with pluripotency and the formation of the three germ layers in the RNA‐Seq data (Figure 4). Ts1Cje‐mESC‐derived cells on differentiation day 4 expressed higher mRNA levels of pluripotency markers, including *Eras*, *Fgf4*, *Gdf3*, *Kit*, *Myb*, *Nanog*, *Nr0b1*, *Pou5f1*, *Tdgf1* and *Zic3* [35] in comparison to wild‐type mESCs (Figure 4A), suggesting the persistence of pluripotency in Ts1Cje‐mESCs. Furthermore, mRNA expression levels of mesoderm differentiation markers such as *Evx1*, *Hand1*, *Snai1*, *Snai2*, *T*, *Tal1*, *Tbx20*, *Tbx4*, *Twist1*, and *Twist2* [36, 37, 38, 39] were reduced in Ts1Cje‐mESC‐derived cells (Figure 4A). Interestingly, the mRNA levels of endoderm differentiation markers *Afp*, *Cldn6*, *Foxa1*, *Foxa2*, *Hnf1b*, *Hnf4a*, *Sox17* and *Ttr* [38, 40, 41, 42] were increased in Ts1Cje‐mESC‐derived cells. However, no differences in the mRNA levels of ectoderm differentiation markers, such as *Ascl1*, *Dtx4*, *Foxj3*, *Hes5*, *Neurog1*, *Pax2*, *Sox1*, *Tubb3* and *Zic1* [39, 43] were detected between Ts1Cje‐mESC‐ and wild‐type mESC‐derived cells (Figure 4A).

In our experimental procedure to induce primitive macrophages, cytokines such as BMP4 and VEGF were added to the culture medium and induced in the mesoderm to produce primitive macrophages. To analyse the spontaneous effect of triplicated genes of Ts1Cje mice on fate decisions using a simplified approach, embryoid body formation assays were performed using Ts1Cje‐mESCs under cytokine‐free conditions [44]. Ts1Cje‐mESC‐derived cells cultured for 14 days in cytokine‐free medium exhibited the substantially decreased expression of *Sox1* mRNA, an ectoderm marker, in comparison to wild‐type cells. In contrast, *Sox17*, an endodermal marker, was significantly upregulated in Ts1Cje‐mESC‐derived cells (Figure 4B). The expression of *T*, a mesoderm marker expressed in the notochord, decreased significantly, whereas *Hand1*, a mesoderm marker expressed in cardiac tissue, tended to increase in Ts1Cje‐mESC‐derived cells (Figure 4B).

Based on the hypothesis that differentiation abnormalities might be attributable to alterations in the cytokine receptor expression, we generated MA plots from the RNA‐Seq data and focused on changes in the expression of genes encoding cytokine receptors during mesoderm differentiation. (Figure 4C). The MA plot shows the base mean and fold change of the gene expression on their logarithmic axes, indicating not only an increase or decrease in gene expression but also the abundance of the gene expression. This plot revealed that *Bmpr1b* and *Flt1*, which encode receptors for BMP and VEGF, were significantly increased by approximately 2‐fold in Ts1je‐mESC‐derived cells in comparison to wild‐type cells. However, the MA plot indicated a relatively low expression of *Bmpr1b* in wild‐type mESC‐derived cells; therefore, the effects of an approximately 2‐fold increase in the *Bmpr1b* expression in Ts1je‐mESC‐derived cells are assumed to be limited. In contrast to *Bmpr1b*, the expression levels of *Flt1*, which encodes VEGFR1, were abundant even in wild‐type mESC‐derived cells; therefore, the further upregulation in Ts1Cje‐mESC‐derived cells may not be relevant to the disruption of cytokine signalling. On the other hand, cytokine receptors *Kdr* and *Flt4* were found in the MA plot analysis as decreased genes in Ts1Cje‐mESC‐derived cells. These genes are involved in mesoderm differentiation and are highly expressed in wild‐type mESC‐derived cells. In addition, *Kdr* and *Flt4* were markedly and slightly downregulated, respectively, in Ts1Cje‐mESC‐derived cells (Figure 4C). As *Kdr* and *Flt4* encode VEGFR2 and VEGFR3, which promote mesodermal angiogenesis and haematopoiesis [27, 31], the significant downregulation of these genes may directly induce the suppression of mesodermal differentiation in Ts1Cje‐mESCs. To further validate the changes in the expression of cytokine receptors at the protein level, flow cytometry revealed that the expression levels of BMPR1B and VEGFR1 showed a slight increasing trend, whereas VEGFR2 was significantly decreased in Ts1Cje‐mESC‐derived cells (Figure 4D). In contrast, the expression of VEGFR3 did not change between the genotypes (Figure 4D).

### 3.4 Possible Activation of Inflammatory Pathway in Primitive Macrophages Derived From Ts1Cje‐mESCs

**FIGURE 5:**
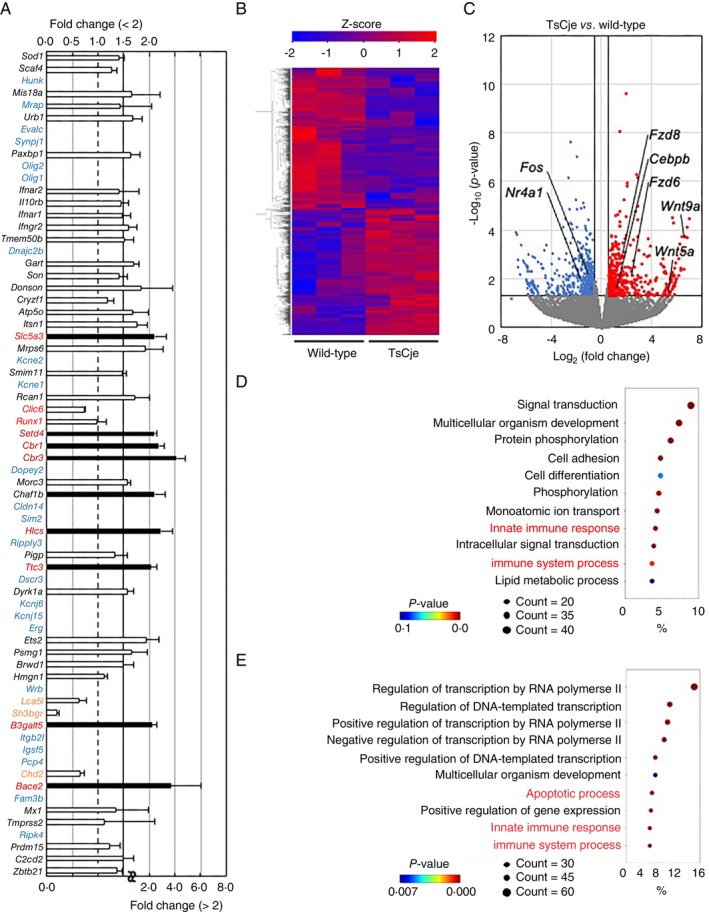
Pro‐inflammatory and neurodegeneration‐related genes were up‐regulated in primitive macrophages derived from Ts1Cje‐mESCs. **(A)** Expression change of genes in the trisomic region of Ts1Cje mice. Genes with a fold change < 2 are represented by white bars (corresponding to the upper axis), and genes with a fold change > 2 are represented by black bars (corresponding to the lower axis). The colours of the genes, black, red, brown and blue, indicate approximately 1.5‐fold change, more than 2‐fold upregulation, downregulation, and no expression, respectively (*n* = 3 for each group). Data presented an overview of expression trends as the mean ± SEM from three independent biological replicates. (B) Heatmap of genes with *p* value < 0.05 and fold change > 3/2 or < 2/3 in the RNA‐Seq dataset of primitive macrophages derived from wild‐type‐ and Ts1Cje‐mESCs. The colours of the plots, red and blue, indicate significant upregulation and downregulation, respectively (*n* = 3 for each group). Data presented from three independent biological replicates. Statistical analysis was performed using Wald test and adjusted using Benjamini–Hochberg correction. (C) Volcano plot is drawn with the RNA‐Seq dataset. (D, E) GO analysis of DEGs. The GO enrichment analysis of DEGs upregulated (D) and downregulated (E) in Ts1Cje‐mESC‐derived cells was retrieved using the DAVID program. Top 10 enriched GO terms in biological process are presented (*n* = 3 for each group). Data presented from three independent biological replicates. Statistical analysis was performed using Fisher's exact test.

On differentiation day 12, primitive macrophages (CD45^+^/F4/80^+^ cells) arising from the wild‐type and Ts1Cje‐mESCs were sorted (Figure S4A, purity > 90%) and profiled using bulk RNA‐Seq. In the PCA results, separated clusters were detected, although they were not as distinct as in the case of cells on differentiation day 4 (Figure S4B). Many genes in the trisomic region of Ts1Cje mice were upregulated in a gene dose‐dependent manner in primitive macrophages derived from Ts1Cje‐mESCs on day 12 (black text in Figure 5A). Furthermore, the expression levels of the nine trisomic genes *Slc5a3*, *Sted4*, *Cbr1*, *Cbr3*, *Chaf1b*, *Hlcs*, *Ttc3*, *B3galt5* and *Bace2* were significantly increased by more than 2‐fold in primitive macrophages derived from Ts1Cje‐mESCs in comparison to those derived from wild‐type mESCs (red text in Figure 5A). In contrast, the expression of five trisomic genes (*Clic6*, *Runx1*, *Lca5l*, *Sh3bgr* and *Chd2*) decreased in the primitive macrophages derived from Ts1Cje‐mESCs in comparison to those derived from wild‐type mESCs, and there were 22 triplicated genes that were undetectable (brown and blue text in Figure 5A, respectively).

Gene expression profiling showed 987 DEGs containing 466 upregulated genes and 521 downregulated genes in primitive macrophages derived from Ts1Cje‐mESCs, defined as *p* value < 0.05, and fold change > 3/2 or < 2/3, respectively (Figure 5B,C). The upregulated genes included *Cebpb*, which drives pro‐inflammatory and neurodegeneration‐related gene programs [45, 46], and *Wnt5a*, *Wnt9a*, *Fzd6* and *Fzd8*, factors related to Wnt signalling, which increase the expression of the pro‐inflammatory immune response genes in macrophages (Figure 5C) [47, 48, 49, 50]. In contrast, the downregulated genes included *Fos* and *Nr4a1*, which suppress microglial activation and the expression of pro‐inflammatory genes (Figure 5C) [51, 52, 53].

A GO analysis of both upregulated (Figure 5D) and downregulated DEGs (Figure 5E) showed enrichment in genes related to the innate immune response and immune system processes. These findings are consistent with our previous observation that microglia with activated morphology were observed in the fetal brains of Ts1Cje mice [7]. Interestingly, the genes involved in the apoptotic process were enriched in the upregulated genes on differentiation day 4 (Figure 3D), whereas they were enriched in the downregulated genes on differentiation day 12 (Figure 5E).

**FIGURE 6:**
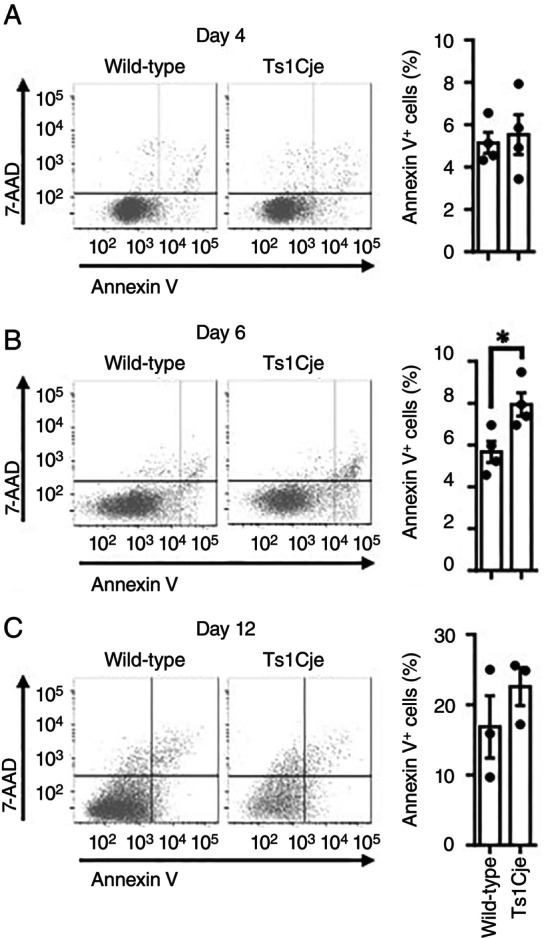
Increased apoptosis was more pronounced in Ts1Cje‐mESC‐derived cells than in wild‐type. (A) Comparable proportion of the apoptotic cell population in wild‐type and Ts1Cje‐mESC‐derived cells on differentiation day 4. (B) Increased proportion of apoptotic population in Ts1Cje‐mESC‐derived cells in comparison to wild‐type on differentiation day 6. (C) Comparable proportion of apoptotic cell population in wild‐type and Ts1Cje‐mESC‐derived cells on differentiation day 12. (A–C) Apoptotic cells were detected by flow cytometry (*n* = 4 (A, B), *n* = 3 (C) for each group). Data are presented as the mean ± SEM from four (A, B), three (C) independent biological replicates. Statistical analysis was performed using a two‐tailed unpaired *t*‐test. Not significant (*p* = 0.5714 (A), *p* = 0.3348 (C)); *p < 0.05 (*p* = 0.0240 (B)).

Apoptotic cells were assessed using Annexin V. As shown in Figure 6A–C, increased apoptotic cell death was detected in Ts1Cje‐mESC‐derived cells on differentiation day 6 (Figure 6B) but not on day 4 (Figure 6A). On day 12, the apoptosis of Ts1Cje‐mESC‐derived primitive macrophages was comparable to that of wild‐type macrophages (Figure 6C).

### 3.5 Decreased Number of Primitive Macrophages in the Yolk Sac of Ts1Cje Mice

**FIGURE 7:**
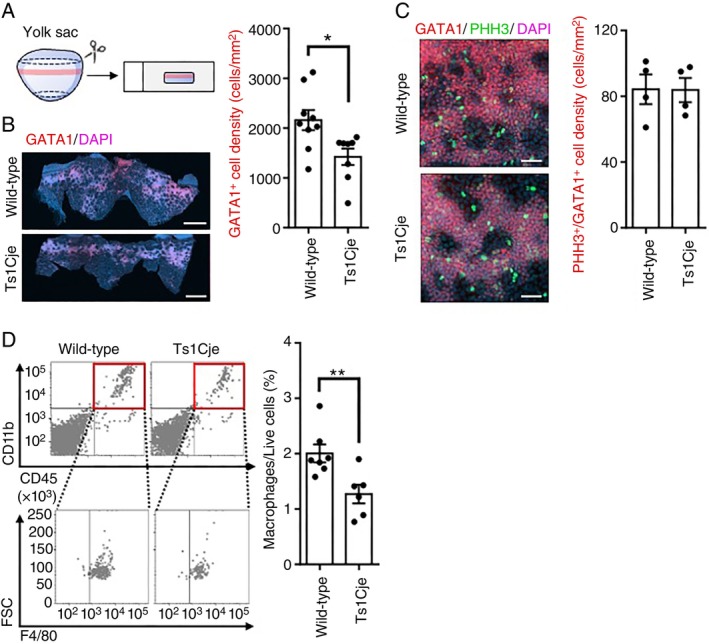
Primitive macrophages and their progenitors decreased more in the yolk sac of Ts1Cje mice than in wild‐type ones. (A) Schematic diagram of the dissection of the murine yolk sac (E8.5). (B, C) Decreased ratio of GATA1^+^ cells including precursors of primitive macrophages in the Ts1Cje yolk sac. The GATA1‐expressing cells in yolk sacs of wild‐type and Ts1Cje mice at E8.5 were detected by whole‐mount GATA1 immunofluorescence staining. Proliferating cells were assessed by PHH3 immunofluorescence staining. Scale bars are 500 μm (B) and 50 μm (C). Data are presented as the mean ± SEM, *n* = 9 for wild‐type mice and *n* = 8 (B) for Ts1Cje mice, *n* = 4 for each group (C). Outliers were identified and excluded from statistical analysis using Grubbs's test (*α* = 0.05). Statistical analysis was performed using a two‐tailed unpaired *t*‐test. Not significant (*p* = 0.9719 (C)); *p < 0.05 (*p* = 0.0164 (B)). (D) Decreased number of primitive macrophages in the yolk sac of wild‐type and Ts1Cje mice at E8.5. Primitive macrophages were analysed by flow cytometry, and are defined as CD45^+^, CD11b^+^ and F4/80^+^ cells. The graph shows the percentages of primitive macrophages from the total live cells in the yolk sac. Data are presented as the mean ± SEM, *n* = 7 for wild‐type mice and *n* = 6 for Ts1Cje mice. Statistical analysis was performed using a two‐tailed unpaired *t*‐test. **p < 0.01 (*p* = 0.0091). All samples were obtained from littermates as biological replicates.

In a previous study, primitive macrophages were found to be populated in yolk sacs of E8.5 embryos, but no primitive macrophages were detected in the brain primordium at this time point [3]. We examined the number of primitive macrophages in the yolk sac of Ts1Cje mice at E8.5 to elucidate the inhibitory effect of Ts1Cje trisomic genes on the production of primitive macrophages in vivo. Since GATA1^+^ cells are precursors of primitive macrophages [54], the yolk sac from E8.5 foetus was immunohistochemically stained with an anti‐GATA1 antibody. As shown in the schematic diagram (Figure 7A), anti‐GATA1 whole‐mount‐stained yolk sacs were mounted on glass slides and analysed using laser confocal microscopy (Figure 7B,C). In Ts1Cje embryos, the area occupied by GATA1^+^ cells in the yolk sac was significantly decreased in comparison to that in wild‐type littermates (Figure 7B). Phospho‐histone H3 (PHH3) is a marker of cell proliferation. Additionally, the ratio of PPH3^+^ cells among GATA1^+^ cells showed no difference between the two groups (Figure 7C), suggesting that processes other than cell proliferation are involved in reducing primitive macrophage progenitor cells. Furthermore, flow cytometry demonstrated that the proportion of primitive macrophages (CD45^+^/CD11b^+^/F4/80^+^) in the yolk sac of Ts1Cje mouse embryos at E8.5 was also significantly lower than that in wild‐type littermates (Figure 7D).

### 3.6 Inhibited Differentiation of hiPSCs With Trisomy 21 Towards Primitive Macrophages

**FIGURE 8:**
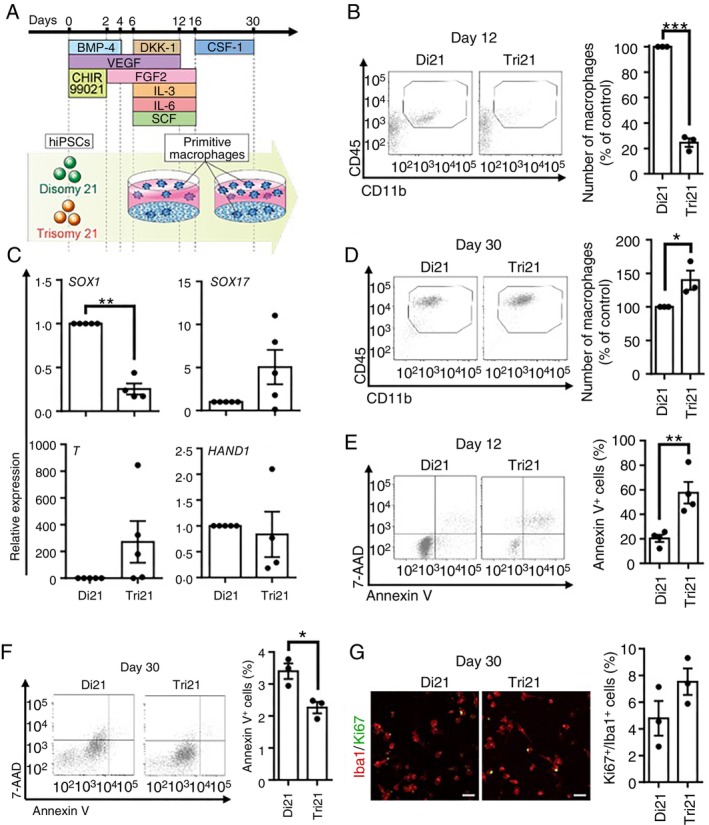
Trisomy 21 disturbed differentiation into primitive macrophages from hiPSCs. (A) Schematic diagram of the schedule for differentiation of hiPSC into primitive macrophages. (B) Decreased number of primitive macrophages in hiPSCs with trisomy 21 (Tri21) in comparison to hiPSCs with disomy 21 (Di21) on differentiation day 12. The primitive macrophages were defined as CD45^+^/CD11b^+^ cells on flow cytometry. Graphs show the total number of primitive macrophages calculated as percentage of Di21 (*n* = 3 for each group). Data are presented as the mean ± SEM from three independent biological replicates. Statistical analysis was performed using two‐tailed unpaired *t*‐test. ***p < 0.001 (*p* < 0.0001). (C) Disturbance of fate direction of Tri21‐hiPSCs in the cytokine‐free culture medium. Expression levels for three germ layers markers were analysed by RT‐qPCR in an embryonic body for 14 days (*n* = 5 for each group). Outliers were identified and excluded from the statistical analysis using Grubbs's test (*α* = 0.05). Data are presented as the mean ± SEM from five independent biological replicates. Statistical analysis was performed using two‐tailed one sample *t*‐test. Not significant (*p* = 0.1126 *SOX17*, *p* = 0.1568 *T, p* = 0.7330 *HAND1*); **p < 0.01 (*p* = 0.0013 *SOX1*). (D) The number of primitive macrophages derived from Tri21‐hiPSCs was increased in comparison to those from Di21‐hiPSCs on long‐term culture, differentiation day 30. The primitive macrophages were defined as CD45^+^/CD11b^+^ cells on a flow cytometric analysis. Graphs show the total number of primitive macrophages calculated as the percentage of Di21 (*n* = 3 for each group). Data are presented as the mean ± SEM from three independent biological replicates. Statistical analysis was performed using two‐tailed unpaired *t*‐test. *p < 0.05 (*p* = 0.0480). (E, F) The proportion of apoptotic cells from Tri21‐hiPSCs was increased in comparison to that from Di21‐hiPSCs on differentiation day 12 but not on day 30. Apoptotic cells on day 12 (E) and day 30 (F) were detected by flow cytometry (*n* = 4 (E), *n* = 3 (F)). Data are presented as the mean ± SEM from four (E) and three (F) independent biological replicates. Statistical analysis was performed using two‐tailed unpaired *t*‐test. *p < 0.05 (*p* = 0.0194 (F)); **p < 0.01 (*p* = 0.0071 (E)). (G) No significant difference in the proliferation of macrophages between Di21‐ and Tri21‐hiPSCs on day 30. Proliferating cells and macrophages were detected by immunostaining with anti‐Ki67 and anti‐Iba1 antibodies, respectively (*n* = 3 for each group). Scale bars are 50 μm. Data are presented as the mean ± SEM from three independent biological replicates. Statistical analysis was performed using two‐tailed unpaired *t*‐test. Not significant (*p* = 0.1677). The Iba1^+^ cells (Di21: 189 cells and Tri21: 104 cells) were analysed. BMP‐4, bone morphogenic protein‐4; CSF‐1, colony stimulating factor 1; Di21, disomy 21; DKK1, dickkopf‐1; FGF2, fibroblast growth factor 2; hiPSCs, human induced pluripotent stem cells; IL‐3, interleukin‐3; IL6, interleukin‐6; SCF, stem cell factor; Tri21, trisomy 21; VEGF, vascular endothelial growth factor.

Finally, to determine whether abnormalities in the production of primitive macrophages caused by trisomic genes were also observed in humans with DS, primitive macrophages were induced from an hiPSC line established from an individual with trisomy 21 (HPS4270) and an isogenic diploidized hiPSC line, in which a maternal HSA21 was derived from the HPS4270 cell line (HPS4272) [16]. In this protocol, generated primitive macrophages were collected as floating cells (Figure 8A). As with mESCs, among the floating cells collected on differentiation day 12, the number of primitive macrophages, defined as CD45^+^/CD11b^+^, was significantly reduced in hiPSCs with trisomy 21 (Tri21‐hiPSCs)–derived cells in comparison to that in diploidized hiPSC‐derived cells (Figure 8B). We also assessed the ability of Tri21‐hiPSCs to differentiate into three germ layers using a simplified approach. An EB formation assay in cytokine‐free medium demonstrated a significant decrease in the ectoderm marker *SOX1* and a tendency to increase in the endoderm marker *SOX17* in Tri21‐hiPSCs in comparison to hiPSCs with disomy 21 (Di21‐hiPSCs) (Figure 8C). The expression of the tissue‐specific mesodermal markers *T* and *HAND1* showed increasing and decreasing trends in Tri21‐hiPSC‐derived cells, respectively, relative to Di21‐hiPSCs (Figure 8C).

The protocol for inducing primitive macrophages from hiPSCs also produces adherent cells as feeder cells, which makes it possible to culture primitive macrophages for longer periods than the mouse stem cell protocol [12]. When further longer‐term culture, such as for 30 days, was performed, the total number of primitive macrophages (CD45^+^/CD11b^+^) derived from Tri21‐hiPSCs significantly increased in comparison to those derived from Di21‐hiPSCs (Figure 8D).

We further analysed apoptosis using Annexin V, as with mESCs. The proportion of apoptotic cells among Tri21‐hiPSC‐derived cells (CD45^+^) was significantly higher than that among Di21‐hiPSCs on day 12 (Figure 8E). In contrast, the proportion of apoptotic Tri21‐hiPSC‐derived cells decreased by day 30. However, the extent of this decrease was small (Figure 8F). Additionally, immunostaining for Ki67 revealed no significant difference in the proliferative capacity between trisomy Tri21‐ and Di21‐hiPSC‐derived macrophages on day 30 (Figure 8G). These results suggest that the increased number of Tri21‐hiPSC‐derived macrophages is not attributable to enhanced cell proliferation.

## 4 Discussion

It has been reported that microglia are activated during prenatal and postnatal life in the brains of DS model mice [7, 55]. Similarly, in the postmortem human brain with DS, microglial activation has been evidenced by increased levels of inflammatory cytokines [56]. However, no studies have been conducted on abnormalities in the microglial differentiation process in DS. This study demonstrated that triplicated genes associated with DS induce a gene expression profile indicative of inflammatory activation in primitive macrophages, which are precursors to microglia. Furthermore, for the first time, this study revealed that triplicated genes in DS inhibit the differentiation of these primitive macrophages both in vitro and in vivo. These findings help clarify our earlier observations of morphologically activated and reduced numbers of microglia in the brains of Ts1Cje mice at E14.5 [7].

This study utilising Ts1Cje‐mESCs and Tri21‐hiPSCs revealed several important findings regarding the disturbed differentiation of progenitors into the three germ layers in DS. It demonstrated not only impaired differentiation into the mesoderm, which includes the differentiation of primitive macrophages, but also a tendency to preferentially differentiate towards the endoderm while suppressing differentiation towards the ectoderm in DS. Additionally, neuroectodermal differentiation was shown to be inhibited in the DS model of mESCs containing a single copy of HSA21 [57]. This observation is consistent with our results showing the decreased expression of *Sox1* mRNA, an ectodermal marker, in Ts1Cje‐mESCs and Tri21‐hiPSCs. Regarding preferential differentiation towards the endoderm, neural progenitor cells induced from Tri21‐hiPSCs have been reported to express higher levels of the endoderm marker *AFP* relative to those induced from control hiPSCs [58]. Additionally, malformations of the mesodermal cardiovascular system and endodermal gastrointestinal system are the most common in DS [59], suggesting that the disturbed differentiation towards mesoderm and endoderm caused by trisomy genes demonstrated in this study may be associated with these malformations.

In the present study, we found that cells derived from Ts1Cje‐mESCs exhibited increased apoptotic cell death during differentiation towards the mesoderm. Additionally, it has been suggested that neural progenitor cells derived from Tri21‐hiPSCs also undergo apoptosis because of the triplication of the *RUNX1* gene [60]. In our study, the *Runx1* gene was located within the triplicated region of Ts1Cje‐mESCs and was overexpressed on day 4 of differentiation into primitive macrophages. Therefore, the triplication of the *Runx1* gene may contribute to the increased apoptotic cell death observed under mesodermal conditions. We believe that this induction of apoptosis is a key factor in the reduction of primitive macrophages in the yolk sac of Ts1Cje mice, as demonstrated in our findings. Therefore, given that primitive macrophages are the exclusive source of microglia, their reduction likely accounts for at least part of the decrease in microglial numbers in Ts1Cje embryos, as reported in a previous study [7]. The present study focused on the development of primitive macrophages; thus, we did not investigate the differentiation into other lineages. However, a comprehensive examination of abnormalities in the direction of differentiation is an interesting subject for the future.

The differentiation of Tri21‐hiPSCs into primitive macrophages was disrupted in a pattern similar to that observed in Ts1Cje‐mESCs. Interestingly, we discovered that long‐term culture of primitive macrophages derived from Tri21‐hiPSCs reduced the increase in apoptosis. This suggests that genes associated with trisomy may play a role in apoptotic resistance in differentiated primitive macrophages. In contrast, during long‐term culture, there was no marked difference in proliferation between Tri21‐hiPSC‐ and Di21‐hiPSC‐derived macrophages, indicating that the increased number of Tri21‐hiPSC‐derived macrophages was not attributable to enhanced proliferation. Instead, a slight but significant reduction in apoptosis may have contributed to the increased cell numbers observed in Tri21‐hiPSC‐derived macrophages. Unfortunately, this phenomenon could not be confirmed in Ts1Cje mESCs, because primitive macrophages derived from mESCs cannot be maintained for an extended period. However, the reduction in microglia observed in Ts1Cje foetuses was relatively minor (approximately 40%) [10] compared with the more significant decrease in primitive macrophage production noted in this in vitro analysis (approximately 80% in the present study). Microglia are known for their strong ability to self‐proliferate [61], and even if their numbers drop to just 0.1% in the brain, they can multiply to their original levels within 2 weeks [62]. Therefore, the slight but notable resistance to apoptosis in differentiated primitive macrophages due to trisomy genes may support a compensatory proliferative function of these macrophages in the fetal brain, leading to a minimal reduction in the overall number of microglia in Ts1Cje mice.

This discovery offers new insights into the development of DS foetuses; however, our study has some limitations. First, unlike hiPSCs, mESC‐derived primitive macrophages cannot be maintained in long‐term culture. As a result, whether the observed reduction of the number of primitive macrophages is sustained or transient, and whether these findings are consistent with hiPSC‐derived primitive macrophages is unclear. Second, the conclusions drawn from hiPSC experiments are based on a single cell line. To ensure robustness and reproducibility, future studies using additional cell lines are necessary. Finally, confirming our findings in human brains with DS was challenging due to the limited availability of autopsy brains with DS. However, this issue could be further addressed using advanced human‐based systems, such as brain organoids derived from hiPSCs, in the future [63].

In conclusion, gene expression profiling during the differentiation of Ts1Cje‐mESCs into primitive macrophages revealed several key findings. These cells retained pluripotency, demonstrated resistance to mesodermal differentiation, showed a preference for endoderm differentiation, exhibited vulnerability due to apoptosis, and expressed pro‐inflammatory genes. Additionally, in the yolk sac of Ts1Cje mice, the numbers of primitive macrophages and their progenitor cells were significantly lower than those in the yolk sac of wild‐type mice. An analysis involving Tri21‐hiPSCs also indicated a preference for endodermal differentiation and susceptibility to apoptosis, mirroring the results observed with Ts1Cje‐mESCs. Furthermore, primitive macrophages derived from Tri21‐hiPSCs were able to suppress the increase in apoptosis during long‐term culture, suggesting that the reduction in cell number was transient. These findings provide valuable insights into abnormalities in brain development associated with DS.

## Author Contributions

**Koki Harada:** data curation, formal analysis, validation, investigation, visualisation, methodology, project administration, writing – original draft, review, and editing. **Keiichi Ishihara:** conceptualization, resources, funding acquisition, investigation, validation, methodology, project administration, writing – original draft, review, and editing. **Sayaka Wakayama** and **Teruhiko Wakayama:** investigation and writing – original draft, review, and editing. **Sae Yamamoto:** data curation, writing – review and editing. **Haruhiko Sago:** resources, writing – review and editing. **Shun Shimohama**, **Satoshi Akiba**, and **Florent Ginhoux:** investigation, validation, writing – original draft, review, and editing. **Kazuyuki Takata:** funding acquisition, supervision, investigation, validation, methodology, project administration, writing, review, and editing.

## Funding

This work was supported by the Smoking Research Foundation, Shimizu Foundation for Immunology and Neuroscience, Kyoto Pharmaceutical University Fund for Collaborative Research, Kobayashi Foundation, MEXT‐Supported Program for the Strategic Research Foundation at Private Universities, and Japan Society for the Promotion of Science (22K07033, 22H04822, 20H03569).

## Conflicts of Interest

The authors declare no conflicts of interest.

## Supporting information


**Figure S1:** G‐band karyotyping of Ts1Cje‐mESCs and hiPSCs with trisomy 21 was performed to assess the quality. (A) Images showing the karyotype of wild‐type‐mESCs (left) and Ts1Cje‐mESCs (right), with an addition on chromosome 12. The arrow indicates a translocated segment of the MMU16 region. (B) Images showing the karyotype of hiPSCs (left) and hiPSCs with trisomy 21 (right).
**Figure S2:** The differentiation into primitive macrophages induced from mESC lines derived from other Ts1Cje mice was analysed. The number of primitive macrophages derived from wild‐type and Ts1Cje‐mESCs was assessed based on the total cell numbers and the percentage of CD45^+^/CD11b^+^/F4/80^+^ cells on differentiation day 12. There are three independent cell lines for each of the wild‐type and Ts1Cje mESCs. Differentiation was performed once for each cell line.
**Figure S3:** The gene expression of primitive macrophage progenitors derived from wild‐type‐mESCs and Ts1Cje‐mESCs on differentiation day 4 was analysed by principal component analysis (PCA). Plots show the distribution of the gene expression from each sample along the first two principal components. The explained variance of the principal component is indicated on the axis titles. *n* = 3 for each group.
**Figure S4:** The gene expression of primitive macrophages derived from wild‐type‐mESCs and Ts1Cje‐mESCs on differentiation day 12 was analysed by PCA. (A) Primitive macrophages derived from wild‐type‐ and Ts1Cje‐mESCs were sorted as CD45^+^/F4/80^+^ cells on differentiation day 12 for a comprehensive gene expression analysis. The purity of primitive macrophages differentiated from wild‐type‐ and Ts1Cje‐mESCs was 92.60% ± 2.07% and 93.51% ± 1.26%, respectively. Data are presented as the mean ± SEM. (B) The PCA was performed on the normalised gene expression values. The PCA plot illustrates the distribution of the gene expression from each sample across the first two principal components. The explained variance of the principal component is indicated on the axis titles. *n* = 3 for each group.
**Table S1:** qPCR primers used in this study.

## Data Availability

The datasets produced in this study are available from the Gene Expression Omnibus: RNA‐Seq data: GSE281248 (data on Day 4; https://www.ncbi.nlm.nih.gov/geo/query/acc.cgi?acc=GSE281248) and GSE281256 (data on Day 12; https://www.ncbi.nlm.nih.gov/geo/query/acc.cgi?acc=GSE281256).
