# Supplementary material for: Preventing Differentiation Towards Primitive Macrophages in Stem Cells With Down Syndrome
Source: Immunology. 2025 Nov 27;177(3):596–612. doi: 10.1111/imm.70070 (PMC12867592; doi:10.1111/imm.70070)
Supplement: Supplementary file 1 — Figure S1: G‐band karyotyping of Ts1Cje‐mESCs and hiPSCs with trisomy 21 was performed to assess the quality. (A) Images showing the karyotype of wild‐type‐mESCs (left) and Ts1Cje‐mESCs (right), with an addition on chromosome 12. The arrow indicates a translocated segment of the MMU16 region. (B) Images showing the karyotype of hiPSCs (left) and hiPSCs with trisomy 21 (right). Figure S2: The differentiation into primitive macrophages induced from mESC lines derived from other Ts1Cje mice was analysed. The number of primitive macrophages derived from wild‐type and Ts1Cje‐mESCs was assessed based on the total cell numbers and the percentage of CD45+/CD11b+/F4/80+ cells on differentiation day 12. There are three independent cell lines for each of the wild‐type and Ts1Cje mESCs. Differentiation was performed once for each cell line. Figure S3: The gene expression of primitive macrophage progenitors derived from wild‐type‐mESCs and Ts1Cje‐mESCs on differentiation day 4 was analysed by principal component analysis (PCA). Plots show the distribution of the gene expression from each sample along the first two principal components. The explained variance of the principal component is indicated on the axis titles. n = 3 for each group. Figure S4: The gene expression of primitive macrophages derived from wild‐type‐mESCs and Ts1Cje‐mESCs on differentiation day 12 was analysed by PCA. (A) Primitive macrophages derived from wild‐type‐ and Ts1Cje‐mESCs were sorted as CD45+/F4/80+ cells on differentiation day 12 for a comprehensive gene expression analysis. The purity of primitive macrophages differentiated from wild‐type‐ and Ts1Cje‐mESCs was 92.60% ± 2.07% and 93.51% ± 1.26%, respectively. Data are presented as the mean ± SEM. (B) The PCA was performed on the normalised gene expression values. The PCA plot illustrates the distribution of the gene expression from each sample across the first two principal components. The explained variance of the principal component is indicated o [file IMM-177-596-s001.docx]

**Supplementary Material**

**Preventing differentiation toward primitive macrophages in stem cells with Down syndrome**

Koki Harada^1^, Keiichi Ishihara^2,^*, Sayaka Wakayama^3^, Teruhiko Wakayama^3^, Sae Yamamoto^2^, Haruhiko Sago^4^, Shun Shimohama^5,6,7^, Satoshi Akiba^2^, Florent Ginhoux^8,9,10,11,12^, Kazuyuki Takata^1^

^1^Joint Research Laboratory, Division of Integrated Pharmaceutical Sciences, Kyoto Pharmaceutical University, Misasagi, Yamashina-ku, Kyoto 607–8414, Japan

^2^Laboratory of Pathological Biochemistry, Division of Pathological Sciences, Kyoto Pharmaceutical University, Misasagi, Yamashina-ku, Kyoto 607–8414, Japan

^3^Advanced Biotechnology Center, University of Yamanashi, Yamanashi 400-8510, Japan

^4^Sanno Birth Center, Akasaka, Minato-ku, Tokyo, 107-0052 Japan

^5^Department of Neurology, Sapporo Medical University, School of Medicine, Sapporo 060-8543, Japan

^6^Jiseikai Dementia Center, Itabashi, 175-0045, Japan

^7^Jiseikai Nerima Takanodai Hospital, Nerima, 177-0033, Japan

^8^Singapore Immunology Network (SIgN), Agency for Science, Technology and Research (A*STAR), Singapore 138648, Singapore

^9^Shanghai Institute of Immunology, Shanghai Jiao Tong University School of Medicine, Shanghai 200025, China

^10^Translational Immunology Institute, SingHealth/Duke-NUS, Academic Medical Center, The Academia, Singapore 169856, Singapore

^11^Gustave Roussy Cancer Campus, Villejuif 94800, France

^12^Institut National de la Santé Et de la Recherche Médicale (INSERM) U1015, Equipe Labellisée—Ligue Nationale contre le Cancer, Villejuif 94800, France

*Correspondence to: Keiichi Ishihara, Ph.D.

Laboratory of Pathological Biochemistry, Kyoto Pharmaceutical University

5 Misasagi, Yamashina-ku, Kyoto 607–8414, Japan

Phone: +81-75-595-4656; Fax: +81-75-595-4759

E–mail: [ishihara@mb.kyoto-phu.ac.jp](mailto:ishihara@mb.kyoto-phu.ac.jp)

**
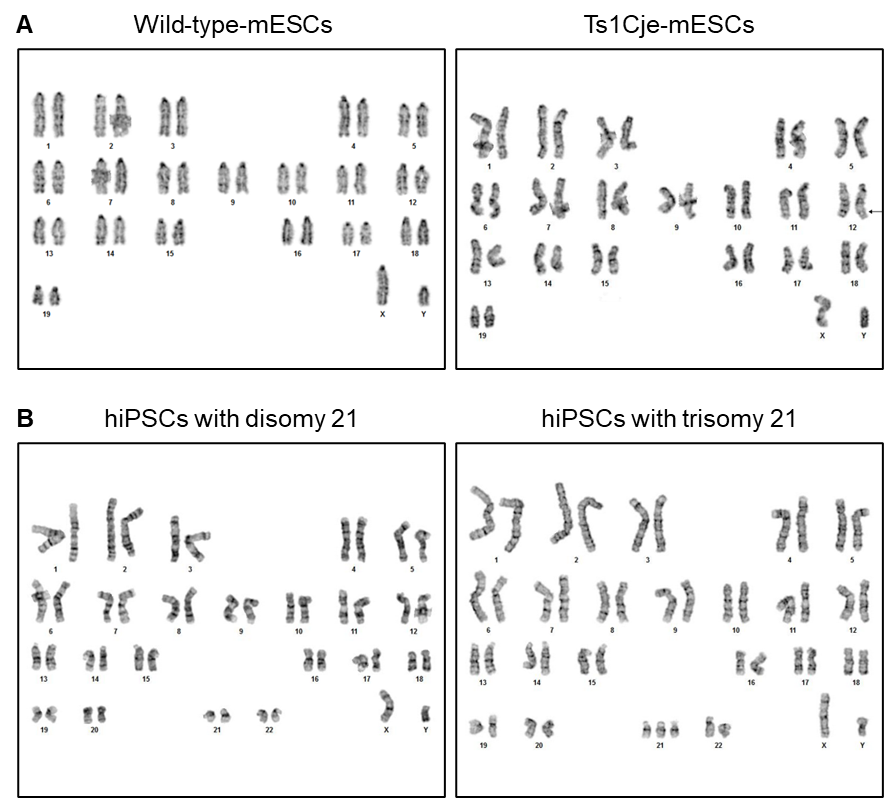
Supplementary Figure S1.** G-band karyotyping of Ts1Cje-mESCs and hiPSCs with trisomy 21 was performed to assess the quality. **(A)** Images showing the karyotype of wild-type-mESCs (left) and Ts1Cje-mESCs (right), with an addition on chromosome 12. The arrow indicates a translocated segment of the MMU16 region. **(B)** Images showing the karyotype of hiPSCs (left) and hiPSCs with trisomy 21(right).
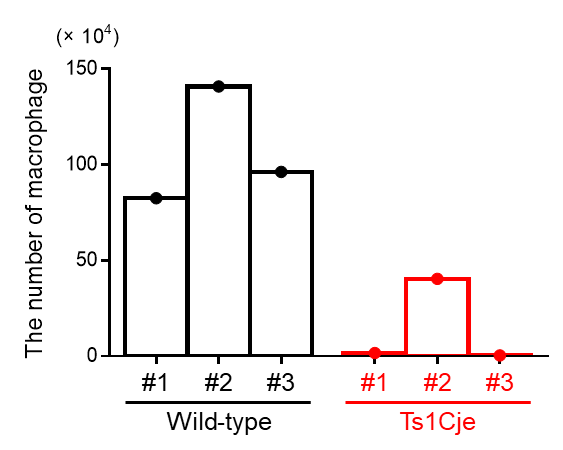
**Supplementary Figure S2.** The differentiation into primitive macrophages induced from mESC lines derived from other Ts1Cje mice was analyzed. The number of primitive macrophages derived from wild-type and Ts1Cje-mESCs was assessed based on the total cell numbers and the percentage of CD45+/CD11b+/F4/80+ cells on differentiation day 12. There are three independent cell lines for each of the wild-type and Ts1Cje mESCs. Differentiation was performed once for each cell line.


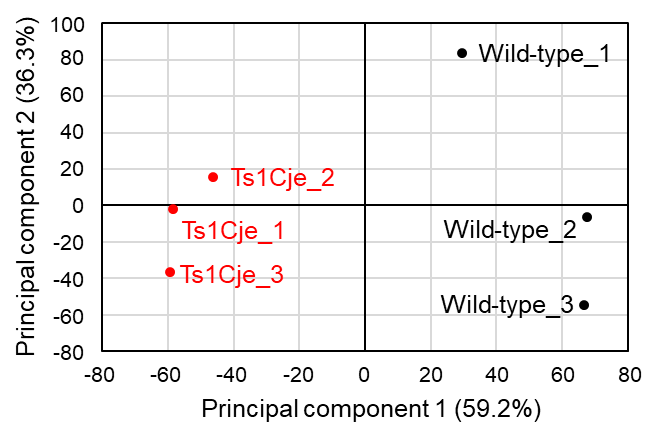
**Supplementary Figure S3.** The gene expression of primitive macrophage progenitors derived from wild-type-mESCs and Ts1Cje-mESCs on differentiation day 4 was analyzed by principal component analysis (PCA). Plots show the distribution of the gene expression from each sample along the first two principal components. The explained variance of the principal component is indicated on the axis titles. *n* = 3 for each group.

**Supplementary
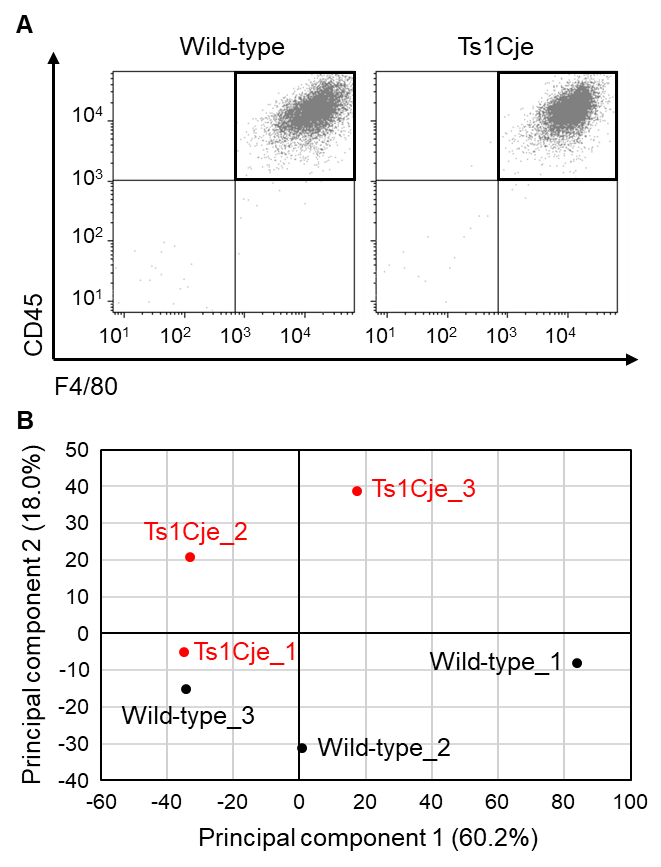
Figure S4.** The gene expression of primitive macrophages derived from wild-type-mESCs and Ts1Cje-mESCs on differentiation day 12 was analyzed by PCA. **(A)** Primitive macrophages derived from wild-type- and Ts1Cje-mESCs were sorted as CD45^+^/F4/80^+^ cells on differentiation day 12 for a comprehensive gene expression analysis. The purity of primitive macrophages differentiated from wild-type- and Ts1Cje-mESCs was 92.60 ± 2.07% and 93.51 ± 1.26%, respectively. Data are presented as the mean ± SEM. **(B)** The PCA was performed on the normalized gene expression values. The PCA plot illustrates the distribution of the gene expression from each sample across the first two principal components. The explained variance of the principal component is indicated on the axis titles. *n* = 3 for each group.

**Supplemental Table S1. qPCR primers used in this study.**

| Gene | Forward (F) or  Reverse (R) | Sequences (5’ – 3’) |
| --- | --- | --- |
| *Sox1* | F | TAGCAGTTTTGAGGCAGCTG |
|  | R | ATCAAAGGCACGCTGTCTTG |
| *T* (mouse) | F | TATGCTCATCGGAACAGCTCTC |
|  | R | ACCAGTTATCATGGGACTGCAG |
| *Hand1* | F | TTCCCCTCTTCCGTCCTCTT |
|  | R | GCAACGAATGGGAACGCTTT |
| *Sox17* | F | TCTAGTGTTGAGGGAAGCAAGG |
|  | R | TCAAAGCCAGCCACAAATGC |
| *Gapdh* | F | AACGACCCCTTCATTGACCTC |
|  | R | ACTGTGCCGTTGAATTTGCC |
| *SOX1* | F | GCGGAGCTCGTCGCATT |
|  | R | GCGGTAACAACTACAAAAAACTTG |
| *HAND1* | F | CTCATTTTCAGCCTTGCCCG |
|  | R | CCCTATTAACGCCGCTCCAT |
| *T* (human) | F | TCACAAAGAGATGATGGAGGAAC |
|  | R | GATGAGGATTTGCAGGTGGA |
| *HAND1* | F | CTCATTTTCAGCCTTGCCCG |
|  | R | CCCTATTAACGCCGCTCCAT |
| *SOX17* | F | CGCTTTCATGGTGTGGGCTAAGGACG |
|  | R | TAGTTGGGGTGGTCCTGCATGTGCTG |
| *GAPDH* | F | *TTGAGGTCAATGAAGGGGTC* |
|  | R | *GAAGGTGAAGGTCGGAGTCA* |
